# Possible Options for Utilization of EU Biomass Waste: Pyrolysis Char, Calorific Value and Ash Content

**DOI:** 10.3390/ma17010226

**Published:** 2023-12-31

**Authors:** Ewa M. Iwanek (nee Wilczkowska), Urszula Nietrzeba, Marta Pietras, Aleksandra Marciniak, Gustaw Głuski, Jakub Hupka, Miłosz Szymajda, Jakub Kamiński, Cezary Szerewicz, Aleksandra Goździk, Donald W. Kirk

**Affiliations:** 1 Faculty of Chemistry, Warsaw University of Technology, Noakowskiego 3, 00-664 Warsaw, Poland; urszula.nietrzeba.stud@pw.edu.pl (U.N.); marta.pietras.stud@pw.edu.pl (M.P.); aleksandra.marciniak2.stud@pw.edu.pl (A.M.); gustaw.gluski.stud@pw.edu.pl (G.G.); jakub.hupka.stud@pw.edu.pl (J.H.); milosz.szymajda.stud@pw.edu.pl (M.S.); jakub.kaminski8.stud@pw.edu.pl (J.K.); cezary.szerewicz.stud@pw.edu.pl (C.S.); aleksandra.gozdzik2.stud@pw.edu.pl (A.G.); 2 Department of Chemical Engineering and Applied Chemistry, University of Toronto, 200 College Street, Toronto, ON M5S 3E5, Canada; don.kirk@utoronto.ca

**Keywords:** biochar, waste crops, energy content, ash composition, volatile compound capture

## Abstract

The application of biomass as a co-feed in coal power plants and in standalone biomass power plants, as well as in char production for soil remediation, is a currently important issue. This paper reports on the investigation of biochar formation from agricultural waste crops that are used for soil upgrading, but which do not meet the standards of EU crops, as well as largescale food processing waste. These were compared to test results from basket willow, which is commonly used for energy generation. Food industry waste is often produced in cities on a large scale and is generally easier to process due to lack of other stream components. The key parameters, namely, the content of volatiles, energy content of the formed biochar and the composition of the ash, were determined for a number of herbaceous materials locally available in the European Union. All of them can be used as a cheap source of biochar. A novel procedure of capturing volatiles and hence minimizing the PAH content in the biochar, as well as enabling the recovery of energy from the volatiles is presented. Knowledge of the composition and form of elements in ash is very important for designing ash management systems if co-combustion is implemented. The aim of this study was to determine if the types of biomass are better suited for biochar production or energy generation.

## 1. Introduction

The application of waste biomass and biofuel crops in energy generation continues to be the focus of numerous studies [1,2,3]. Direct combustion of biomass, either as a co-feed in coal power plants or in standalone biomass power plants has been shown to provide excellent results [4]. The five largest biomass plants are located in Ironbridge (UK), Alholmenskraft (Finland), Toppila (Finland), Polaniec (Poland) and Kymijarvi (Finland). New biomass plants are consistently built not solely in Europe but all over the world. For example, the China Renewable Energy Industry Development Report 2021 states that “more than 80% of biomass raw materials are used for power generation in China” [5]. Similarly, Canada continues to build powerplants based solely on biomass on top of the already operational ones (there are over 100 such plants, e.g., in Atikokan, Ontario), the largest of which are to be located in Fort St. James and Merritt, in British Columbia [6]. Similarly, the United States, Brazil and Germany are also key contributors to the global power generation from biomass [7,8]. All of these plants use biomass typical for the region because apart from providing adequate calorific value, the plant species used for combustion should be widely available to enable largescale operation [9]. For instance, in China, rice husks are the main biomass feedstock used for energy generation [9,10,11]. In the case of Poland, the economic potential is reported to be the highest for (1) energy crops, (2) solid (dry) waste, (3) wet waste and (4) forestry products [3]. Basket willow (*Salix viminalis* L.) was selected as a potential candidate for such application, based on its plentiful abundance in the humid continental climate found in Poland, rapid rate of growth and high calorific value [12,13].

A viable alternative for the combustion of biomass is biochar synthesis. Biochar can be used as an organic compost to improve crop productivity [14,15] or as a construction material [16,17]. It is known that the conditions and feedstock used for biochar synthesis determine the properties of the produced biochar [18,19]. Nowadays, potential applications of biomass as either a feed in biomass power plants or as a biochar for soil remediation are being developed to reduce dependence on fossil fuels and manage food waste from industrial-scale production [20,21]. Catalytic conversion of biomass waste has also been studied as a method to upgrade it [22,23,24]. A novel way to utilize biomass waste is to use the ash to bind CO_2_, which could potentially lower CO_2_ emissions if performed on a large scale [2]. Several parameters are crucial for determining the potential application of a specific type of biomass. One important variable is the composition and abundance of ash. There are two sources of ash in biomass, namely, “inherent ash”, which is found as salts within the carbonaceous material itself, and “entrained ash”, which comes from either harvest or transport of the biomass [25]. Although the “inherent” ash-forming compounds are distributed evenly throughout the biomass, the “entrained ash” is usually not. This is why both char and ash homogeneity were analyzed using scanning electron microscopy coupled with energy-dispersive X-ray spectroscopy (SEM-EDX), which allowed for spot analysis of the biomass, biochar and ash rather than just obtaining an average value.

The goal of this study is to determine the properties, such as composition, char yield, calorific value, etc., to determine the most beneficial way of utilizing common biomass waste obtained from a small-scale agricultural farms in southeastern Poland and compare them to the results obtained for basket willow, which is commonly used for power generation. The selection of the biomass used for soil upgrading and remediation was based on the experience of local farmers, who implement such natural methods in their practice. Crops that are used for soil upgrading but do not meet the standards of EU crops were also chosen for this study [26]. Oats are commonly used for such purposes, widely available in Poland, and some farmers have developed furnaces for combustion of substandard oats, which cannot be sold as an agricultural product (private communication). However, their potential for energy generation [27] or biochar production [28,29] has not been thoroughly studied. Field bean (*Vicia faba* L.) has been selected as a promising candidate due to its implementation as an arable rotation crop in agriculture, which can fix nitrogen into the soil and help reduce cereal pests and disease [30,31]. Moreover, biomass that is easily available as single-component waste streams from largescale food production was identified as peel byproducts of potato (*Solanum tuberosum*), which have been studied [32,33,34], and parsley root peels (*Petroselinum crispum*), which have not been investigated thus far as a potential source of biochar. When waste is created on a large scale, e.g., in a potato chips manufacturing plant, it does not go back to the farm. Hence, alternative ways of utilization to those commonly applied on farms, such as using it to feed livestock or simply composting it, need to be developed. The growing need for managing potato peel waste (PPW) from the potato industry from the perspective of the growing amount of waste has been reported in comprehensive reviews, such as those by Baum et al. [5], Kot et al. [33], Khanal et al. [34] and Garcia-Garcia et al. [35]. The development of methods for the utilization of waste from the food industry, though challenging [35], has been pinpointed as a way to implement circular economy regulations. 

Apart from energy generation, the alternative way of utilizing the biomass considered in this study was for biochar synthesis. A novel approach to biochar formation was applied in this study, namely, placing the biomass in tightly wrapped aluminum foil used to encapsulate the biomass, which allowed for us to use a flowing stream of ambient air to transport the evolving gases onto a layer of molecular sieves placed downstream of the biomass while avoiding combustion of the biochar. This approach allowed for the capture of the evolving volatiles. The energy contained in the volatiles can be determined by combusting the captured volatiles. In industrial practice, the thermal energy would be used to reduce the amount of external heat needed for the process. The char formed is black, brittle and suitable for application in soil remediation because the volatilized products are carried away by the airflow and condense on molecular sieves and not on the biochar itself.

## 2. Materials and Methods

### 2.1. Sample Selection

The sample selection consisted of a set of widely available biomass wastes with a potential for application in co-firing in coal-based power plants or as standalone biomass feedstocks. The samples were selected as a representative of one of three categories: (1) food industry waste: potato peels (*Solanum tuberosum*) and parsley root peels (*Petroselinum crispum*); (2) agricultural waste: field beans (*Vicia faba)*, low-quality oats (*Avena* L.) and hay, as well as one energy crop, i.e., basket willow (*Salix viminalis*). Samples of basket willow, oats and field beans were collected in the Subcarpathian region in Poland. In principle, potato peels can be utilized on farms as feed for animals; when produced in cities during potato chip or French fry manufacture, the potato waste is not transported back to farms but is considered waste material and hence needs a different way of utilization. This single-component waste stream should make it easier to process for energy or char production due to uniformity of texture, composition, etc. Similarly, although good-quality oats have high value, those selected for this study do not meet the European Union standards and cannot be used for commercial purposes. Hence, there is an opportunity to consider energy or char production as a use for this type of agricultural waste.

### 2.2. Sample Preparation

The importance of drying and the variety of techniques used for biomass drying prior to co-firing with coal was described in [36]. In order to avoid complications associated with the moisture content, all of the samples were dried prior to further processing (90 °C for 4 h). Each biomass sample was first made into smaller pieces (between 1 mm and 3 mm). The dried samples were used for proximate analysis, as well as other characterization measurements. For co-firing experiments, the samples were ground to ensure tight contact between the biomass and carbon and heterogeneity of the mixture. Chemical-grade activated charcoal (Merck; 2 wt.% ash content) was used.

### 2.3. Biochar Formation

**Scheme 1:**
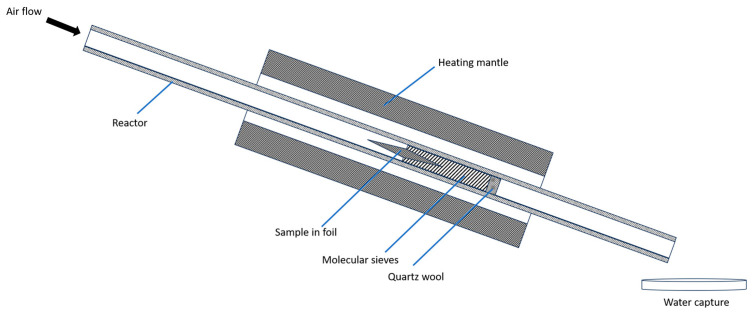
Diagram of the pyrolysis reactor setup.

The prepared samples were tightly wrapped in aluminum foil and placed in a lab-scale quartz glass reactor (10 mm internal diameter). Prior to experiments, the biomass was made into smaller pieces (1–3 mm). The wrapped samples were heated to a temperature of approximately 350 °C for 3 h. An air pump was also connected to the reactor in order to provide a slow rate of airflow through the system. A 6 cm layer of 5A molecular sieves (approx. 5 g) was placed downstream of the sample to capture volatile organic compounds present in outgoing gases. Any water vapor condensate could be readily observed in the back-end of the reactor. A schematic of the reactor is shown in Scheme 1. The weight of samples before and after procedure was noted. The weight loss in the process was attributed to evolution of volatile compounds. The capture of volatiles on the molecular sieves downstream was quantified based on their mass increase. Their calorific value was determined using differential scanning calorimetry coupled with mass spectrometry.

### 2.4. Sample Characterization

The true mean values of the composition of the fresh biomass and biochar samples were determined using elemental analysis. The samples were tested using a CHNS elemental analyzer (Vario EL III instrument, Elementar, Frankfurt, Germany). Each measurement was performed in triplicate. The following gases were used in the analysis: helium (5.0N, Multax, Stare Babice, Poland) and oxygen (5.0N, Linde plc, Dublin, Ireland).

Two types of charring experiments were performed. In the first, the initial charring took place on a hot plate. To ensure that the char was completely devolatilized, the samples were additionally placed in a muffle furnace at 250 °C, 300 °C and 350 °C for 2 h. The other type of charring experiments was performed in a specially designed reactor equipped with an electric, tube-shaped furnace (i.d 1.5 cm). The reactor was tilted at an angle of approx. 20 degrees (Scheme 1). Due to the placement of biomass in aluminum foil, a layer of molecular sieves was placed directly downstream of the aluminum-wrapped biomass. An air pump was used to carry the gases evolving from the samples to be adsorbed and condensed on the molecular sieves.

Ashing was performed by heating the samples over a Mekher burner in a ceramic crucible for 1 h, cooled to room temperature and transferred to a muffle furnace, heated to 700 °C, which was maintained for 1 h. The remaining ash was weighed and analyzed using ATR-FTIR and SEM-EDX. Attenuated total reflectance—Fourier transform infra-red (ATR-FTIR) spectroscopy analysis was performed using a Thermo Fisher Scientific (Dreieich, Germany) Nicolet iS5 instrument equipped with an iD7 ATR accessory with a diamond window. The spectra were collected in the 500–4000 cm^−1^ wavelength range with 16 scans per sample.

The derived biochar and ash were studied using scanning electron microscopy coupled with energy-dispersive X-Ray spectroscopy to determine the topography, heterogeneity and spot composition. Two instruments from Thermo Fisher Scientific (Dreieich, Germany), Prisma E and Helios 5, were used to investigate the composition and elemental distribution. The imaging was performed using Helios 5 (acceleration voltage: 3 kV, ion current: 13 pA, working distance: 4 mm). The energy-dispersive X-Ray spectroscopy measurements were performed using the Prisma E microscope. The parameters used for probing the composition of the individual species and acquiring the elemental maps were acceleration voltage: 15 kV; spot size: 6; working distance: 8 mm. Additionally, experiments with an STEM3+ detector were conducted on Helios 5 using the following parameters: acceleration voltage: 30 kV; ion current: 13 pA; working distance: 4 mm.

### 2.5. Single-Component Combustion and Co-Firing with Carbon

Unlike in the charring experiments, the co-firing measurements were carried out without wrapping the samples. The mixed dry biomass and activated carbon were heated to 850 °C in air so that both the biomass and carbon were fully combusted. The application of thermal analysis in proximate analysis of biomass is a common procedure [37,38]. The experiments were performed on a Netzsch STA 449C Jupiter thermobalance (Netzsch Trockenmahltechnik GmbH, Hanau, Germany) equipped with a quadrupole mass spectrometer, using 4 mg of each sample. The crucible with the sample was equipped with a loose-fitted lid (with a 500 µm hole) to ensure accurate determination of the calorific values. The measurements were performed with a flow (90 mL/min) of synthetic air (80% N_2_, 20% O_2_, Multax, Stare Babice, Poland) and a 10 K/min ramp to 850 °C. Experiments of co-firing were also performed with a 1:1 ratio of biomass and carbon.

## 3. Results

**Figure 1:**
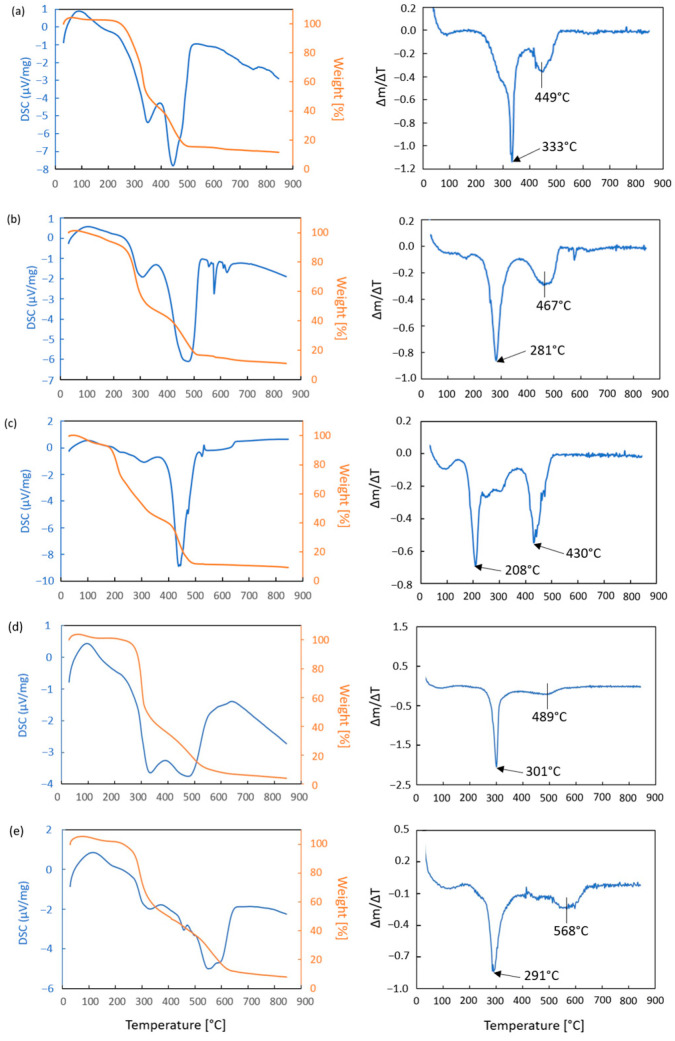
DSC-TGA results obtained for (**a**) basket willow, (**b**) potato peels, (**c**) parsley root peels, (**d**) oats and (**e**) field beans.

When dry biomass is subjected to heating in air or an inert gas to 600 °C, several mass loss steps can be observed [39,40]. First, any remaining moisture is given off by the sample. Next, the sample devolatilizes to form char. During this step, a substantial part of the weight is lost. This is an exothermic reaction regardless of the atmosphere, but the thermal effect is much smaller than that associated with combustion. The evidence for this is that the formed char can be combusted if the sample is further heated in the presence of oxygen. In order to understand the differences in the devolatilization and combustion of each type of biomass and hence evaluate their potential in either biochar preparation or fuel generation, each sample was heated individually in flowing air. The DSC curves depicting the heat generated by the samples (blue) and the weight loss curves (orange) are shown in the left panel of Figure 1.

It can be seen that the devolatilization step of each studied type of biomass is associated with a loss of approximately half of the sample weight. The largest mass loss was noted for parsley root peels, which started weight loss at the lowest temperature, but whose thermal emission was much smaller than from the other types of biomass. Char combustion was typically observed between 400 °C and 500 °C, except for the field beans, which were not fully combusted even at 600 °C. The maximum rate of devolatilization of each sample can be clearly seen in the right panel of Figure 1. This step finished at around 400 °C. The devolatilization of the basket willow exhibited a maximum rate at 330 °C, which is the highest among the studied biomass samples. The lowest temperature was noted for parsley root peels. The other two major parameters that differed between the samples were (1) the difference in the temperatures of maximum rates of devolatilization and char combustion and (2) the relative ratio of the two maxima. The first parameter impacts the possibility of producing char from the biomass and its potential for standalone biomass plants. The difference in the two maximum rates increases in the order willow (116 °C) < potato peels (186 °C) ≈ oats (188 °C) < parsley root peels (222 °C) < field beans (277 °C). This suggests that in the case of field beans, soil remediation may be a better option than energy generation.

The second parameter can be quantified by dividing |Δm/ΔT|_max.dev_ by |Δm/ΔT|_max.comb_. These values correspond to the maximum rate of the devolatilization and char combustion, respectively. The smallest value, i.e., 1.3, was observed for parsley root peels, with the two peaks being very similar. The highest value was 8.0, which was noted for oats. The other three had a similar ratio, which fell in the range 3.0–3.6. The two extreme values can also affect how easy it is to form char from the biomass. This is why biochar was prepared in accordance with two protocols: one in the reactor, in which aluminum foil wrapped around the sample helped to maintain the biomass in the atmosphere of the evolving gases during charring, and the other, which was performed in static air. In the case of static air, charring was conducted at three temperatures: 250 °C, 300 °C and 350 °C. In the case of field beans, 250 °C was too low to convert the biomass to char, whereas 350 °C was too high for charring parsley root peels because partial ashing was observed. Such results of the experiments in static air can be understood when considering the TGA results. The thermogravimetric studies also confirmed that at a temperature of 300 °C all of the types of biomass undergo devolatilization (Figure 1). It is noteworthy that no ashing occurred at 350 °C in the reactor, in which the biochar was produced in the atmosphere of the evolving gases.

**Figure 2:**
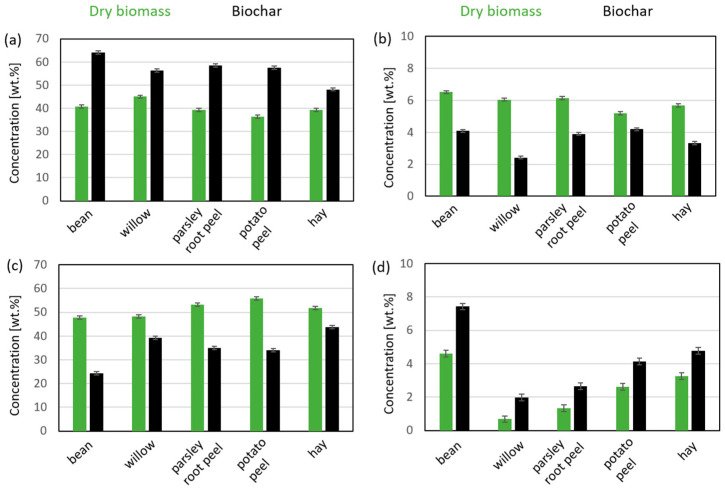
Elemental analysis results: content of (**a**) carbon, (**b**) hydrogen, (**c**) oxygen and (**d**) nitrogen.

Table 1 contains results of the charring and ashing of biomass. The results from the measurements in the pyrolytic reactor at 350 °C are listed in the column on the left and those from experiments carried out in a muffle furnace in static air at 300 °C are on the right. The numbers from the two types of experiments agree for all samples, except for oats, which showed a substantially lower amount of char obtained in static air at 300 °C than in the reactor. This may indicate that the energy released during the devolatilization of oats may be too high to separate the devolatilization from combustion in the presence of oxygen, and this may be the reason why some farmers in Poland have small biomass furnaces for oats (private communication). Indeed, the DSC curve for this type of feedstock confirms that the devolatilization of field beans has a thermal emission of the same magnitude as that of char combustion (Figure 1). Both fresh biomass and biochar derived from it were tested for carbon, hydrogen, nitrogen, oxygen and sulfur content using elemental analysis. All of the results, except those of sulfur, are shown in Figure 2. Sulfur was detected only in fresh, dry field beans, and only 0.4 wt.%. All dry biomass samples contained between 35 and 45 wt.% carbon (Figure 2a) and between 5 and 7 wt.% hydrogen (Figure 2b). The concentration of carbon in the derived biochar ranged between 48 and 65 wt.% and was lowest in the case of hay, whereas it was highest for the field bean char. The hydrogen content decreased to about 4 wt.% in the case of the field bean, parsley root peels and potato peels, whereas it was lowest for the basket willow, i.e., slightly above 2 wt.%. The oxygen concentration in the biomass was 48–52 wt.% (Figure 2c) and decreased during pyrolysis but remained above 20 wt.% for all types of biochar. The decrease in oxygen concentration was largest for the field beans and lowest for hay. The nitrogen concentration varied the most. It was highest in the case of the field beans (4.6 wt.%) and lowest in the case of the basket willow (0.7 wt.%). Such low nitrogen content is in line with the results obtained by Seletnik et. al., who has published several papers and performed thorough studies on basket willow growth in the Subcarpathian Voivodship in Poland [41,42].

**Figure 3:**
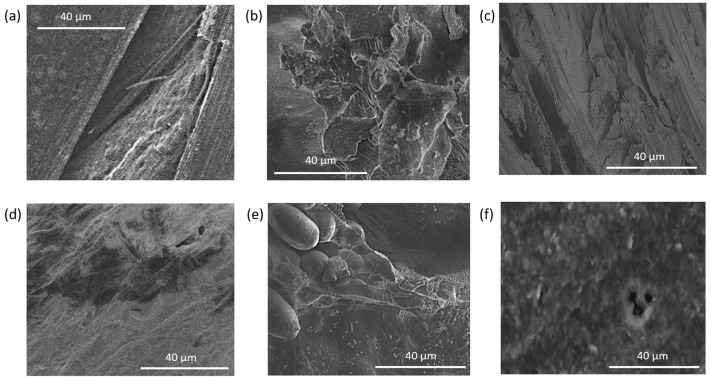
SEM images of biochar samples: (**a**) oats, (**b**) field bean, (**c**) willow, (**d**) parsley root peels, (**e**) potato peels and (**f**) hay.

The topography and heterogeneity of the char can be seen in Figure 3. Different types of char have different morphologies. The biomass charred to maintain the shape of the dry biomass. Upon magnification, it was observed that the char is not homogeneous, especially field beans and potato peels, which have small pieces scattered on the larger particles (Figure 3). Both the charred oats and the willow char have a fibrous appearance. STEM images of potato peel char, oat char and willow char confirm that the difference is also visible at higher magnifications (Figure S1). SEM-EDX measurements were used to probe the char composition. Unlike elemental analysis, this tool cannot determine the concentration of hydrogen. Moreover, it probes the surface of the char, not the entire volume, but this can be beneficial when trying to establish the composition of specific features seen in the images.

**Figure 4:**
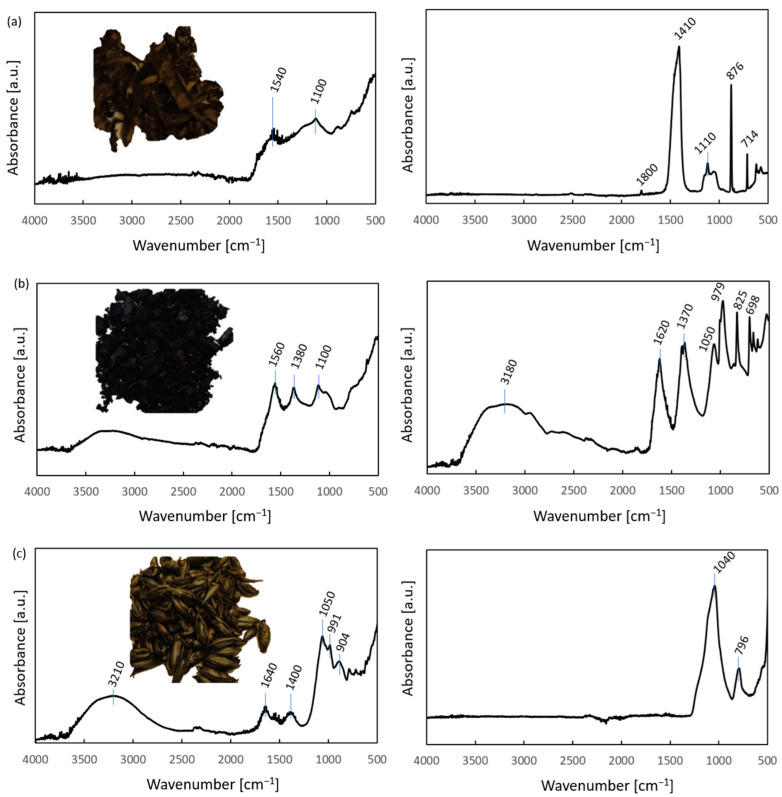
Images and ATR-FTIR spectra of biochar (**left panel**) and ash (**right panel**) derived from (**a**) willow, (**b**) potato peels and (**c**) oats.

Charred samples were analyzed using FTIR-ATR spectroscopy to assess the chemical composition of the obtained product in terms of the presence of C-H stretching bands. A sufficiently charred sample is expected not to have aliphatic C-H bonds, indicated by a lack of corresponding bands in the FTIR spectra as in the case of charred plastics, reported by us previously [43]. Examples of biochar samples obtained from the basket willow, potato peels and oats can be seen in Figure 4. In all cases, the obtained char was black and brittle, but it maintained the shape of the dry biomass. The wide OH band visible in the spectrum is attributed to the hygroscopic nature of all biochars (and ash). The intensity of this band increases with the increase in time between char synthesis and the ATR-FTIR measurement. It can be stated that the ATR-FTIR spectrum of biochar obtained from oats (Figure 4c) is slightly different than those from other feedstocks (Figure 4a,b) in the 1800–1200 cm^−1^ region, which indicates a smaller concentration of the bands from this region in the char. The spectra of different types of biochar contain overlapping bands from both organic and inorganic (ash) components. A comprehensive study of the impact of treatment temperature on the ATR-FTIR spectra components of coals and chars can be found in [44]. In short, the 1261–1251 cm^–1^ band was ascribed to C=O stretching from ethers and etheric oxygen; the 1091, 1031 and 1010 cm^–1^ bands were attributed to ash; whereas bands in the 900–700 cm^–1^ region corresponded due to aromatic HCC (hydrogen–carbon–carbon) rocking vibrations in either simple aromatics or condensed rings. In the case of the studied chars, no aliphatic stretching bands were observed; hence, other types of aliphatic vibrations were excluded.

In contrast to the char spectra, the interpretation of ash spectra is fairly simple because it is common that one inorganic compound dominates the spectrum. This is the case for the willow ash, in which the main band at (1410 cm^−1^, ν_3_ asym CO_3_) and four other bands—876 cm^−1^ (ν_2_ asym CO_3_), 714 cm^−1^ (ν_4_ sym CO_3_), 1800 cm^−1^ (ν_1_+ ν_4_, sym CO_3_) and 1110 cm^−1^ (ν_1_ sym CO_3_)—can be attributed to calcium carbonate. The spectrum of the potato peel ash most resembles that of potassium bicarbonate, e.g., found in the National Institute of Standards and Technology (NIST), U.S. Department of Commerce, database, though it may be a compilation of several phases, particularly in light of the fact that potassium bicarbonate decomposes at temperatures much lower than that used for ashing.

The spectrum obtained in the case of ashed oats contains two distinct bands, which are an indication of the presence of silica: 1040 cm^−1^ (asym Si-O-Si) and 796 cm^−1^ (sym Si-O-Si). Despite such substantial differences in the main components of ash, the overall ash content was below 10 wt.%, though the smallest in the case of oats, but highest for potato peels. Potato peels have relatively large ash content, mainly potassium-containing compounds, which is used by farmers in the Subcarpathian region of Poland for cleaning furnaces of carbon deposits after prolonged operation using other types of biomass (private communication).

**Figure 5:**
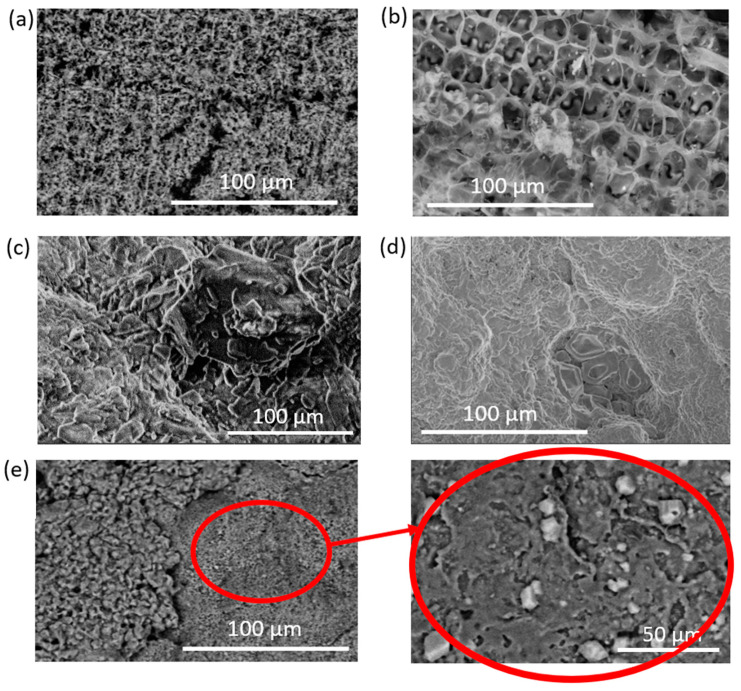
SEM images of ash obtained from: (**a**) willow, (**b**) oats, (**c**) potato peels, (**d**) parsley root peels and (**e**) field beans (a part of the surface was enlarged to show the detailed topography).

The morphology of the ash can be seen in Figure 5. It is very diversified between the samples with particles of different morphology. For instance, the ash from the willow (Figure 5a) is more porous than that obtained from the potato peels (Figure 5c) and parsley root (Figure 5d) peels. The oat ash is the most porous of all the tested ash samples (Figure 5b). The ash from field beans is the most diversified of all (Figure 5e). It can be seen in the image that there are more porous regions (left) and more dense materials on the right, enhanced with even denser particles, which look like well-formed crystals and are more visible on the enlargement of that area. In order to probe the composition of different types of particles from the images, spot EDX measurements were performed.

**Figure 6:**
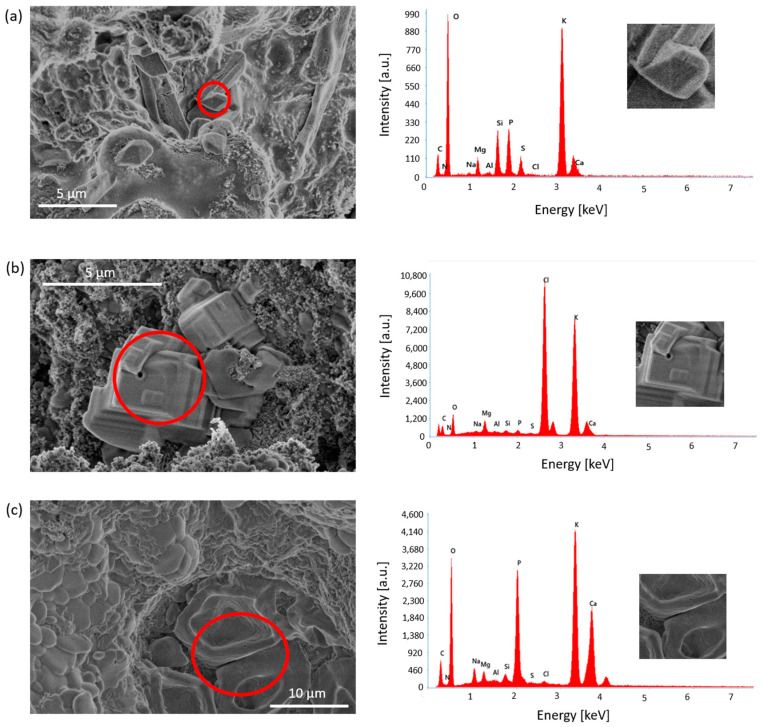
SEM-EDX results of ash from (**a**) hay, (**b**) field bean and (**c**) parsley root peels; the red circle in the image indicates the measurement spot for the obtained spectra.

A benefit of the application of SEM-EDX for this type of sample is that it enables the quantification of much more than just carbon, hydrogen, nitrogen and sulfur. Moreover, the determination of the most likely compounds in which the elements are present is often possible, especially in the case of ash, in which their concentration is higher. The composition of three different crystal forms that differ in composition from the bulk/average compositions can be seen in Figure 6. Although the spectra gathered from the indicated spots contained peaks from the background, the signal of the crystallized compounds were dominant in these spectra and hence allowed for the identification of the phases. The first image contains particles found in hay, which contains very intense signals from potassium and oxygen. This may indicate the presence of potassium oxide or hydroxide, though the latter is more probable to have formed from biomass under the ashing conditions. The carbon signal is too small for carbonate, but both silicon and phosphorus are present in substantial amounts, which may indicate that at least a part of the potassium is in the form of silicate or phosphate. In the case of the crystals found in the field beans (Figure 6b), the particles were large enough to dominate the spectrum to such an extent that the identification of the compound is very easy: KCl. This is interesting because it is a soluble salt and hence can be separated from the non-soluble salts by simple washing. The salt seen in the image of parsley root peel ash (Figure 6c) has signals coming from potassium, calcium, phosphorus and oxygen. This would indicate that the ash contains phosphates of potassium and calcium, or a double salt.

**Figure 7:**
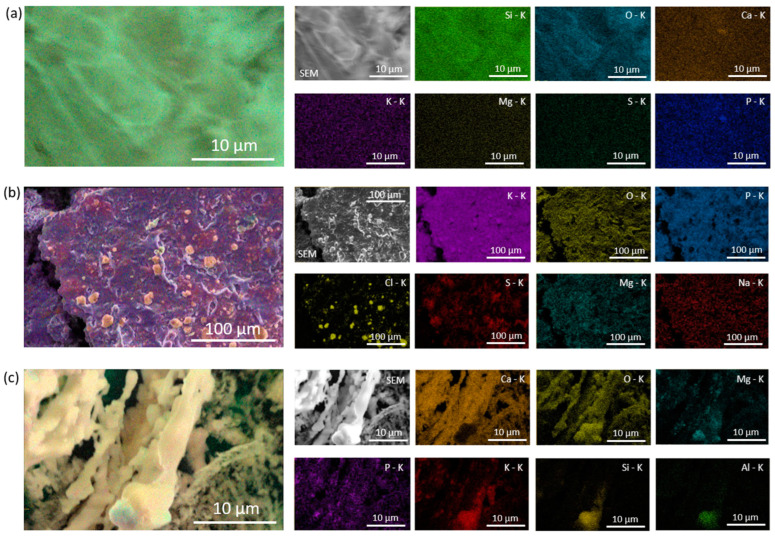
EDX elemental maps of ash (**a**) hay, (**b**) field bean and (**c**) willow.

EDX can also be used for mapping, which is useful in identifying spots enriched in particular elements. Maps of three types of ash are shown as examples: hay ash (Figure 7a), field bean ash (Figure 7b) and willow ash (Figure 7c). It can be seen that each type of the ash is dominated by different elements. In the case of hay, the main components are silicon, oxygen and potassium, which strongly point to potassium silicate. It is noteworthy that calcium and phosphorus are distributed in the same way; hence, they are probably present in the form of one compound, calcium phosphate. The total map for the field bean ash shows that the most abundant element is potassium. The maps that contain “holes” are the background, and those are oxygen and phosphorus. However, the reason that the potassium map contains no “holes” is that the deposit on top of the surface is potassium chloride. Moreover, local enrichment of sulfur was seen on the surface. The third type of ash, willow ash, consists mainly of calcium. Oxygen is also very abundant. This agrees well with the ATR-FTIR spectrum, which shows that calcium carbonate is the main component of willow ash. The distribution of magnesium is similar to that of oxygen. In contrast, phosphorus exhibits a different distribution with additional particles of a phosphorus-containing compound. In the case of willow ash (Figure 7c) enrichments, the main component of ash is calcium and oxygen, although the presence of magnesium and phosphorus was also noted. The local surface enrichments in potassium, silicon and aluminum were similarly distributed.

**Figure 8:**
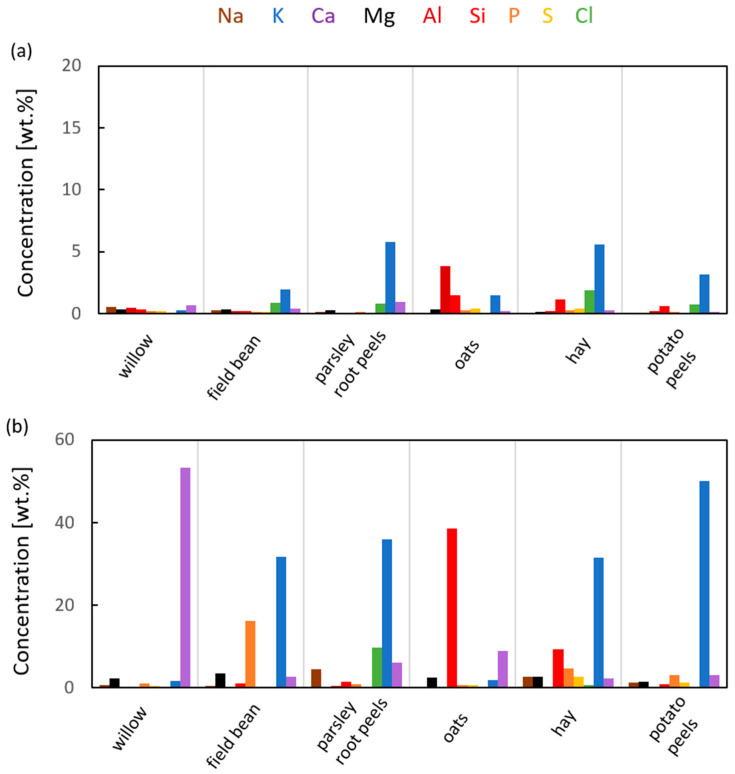
EDX analysis results: the composition of (**a**) biochar and (**b**) ash.

The compilation of the concentration of sodium, potassium, calcium, magnesium, silicon, aluminum, phosphorous, sulfur and chloride for both the biochars and ash is shown in Figure 8. All types of char exhibit a substantial concentration of potassium, except that of the basket willow. The two biochars with the highest potassium concentration are field beans and hay. In the case of oats, aluminum and silicon are also important components of char. When the char is combusted, the composition of ash that remains from oats is dominated by silicon. This is in line with the ATR-FTIR results (Figure 4c), which only shows bands from silica for this ash. Potato peels have the highest concentration of potassium. As seen in other reports, potassium is the most abundant element in ash from this type of biomass; a thorough investigation of the content of different elements in potatoes is provided in [45] and [46]. Potassium ions are known to catalyze the gasification of coal [47], which is why they can catalyze the combustion of carbonaceous deposits in furnaces.

**Figure 9:**
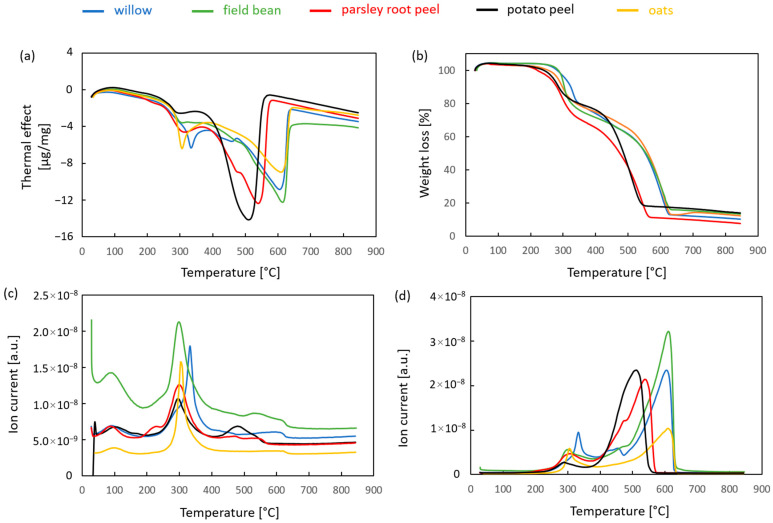
DSC-TGA-MS results of co-firing measurements: (**a**) thermal effect, (**b**) weight loss, (**c**) H_2_O (*m*/*z* = 18) evolution and (**d**) CO_2_ (*m*/*z* = 44) evolution curves.

Co-firing measurements were performed with each of the biomass samples. The results are compiled in Figure 9. They all have similar features, i.e., two exothermic steps, which differ in their intensity and temperature ranges. From the overlay of the DSC curves, it can be seen that the largest exothermic effect is observed for the potato peels (Figure 9a, black curve). The combustion of char and carbon has the lowest maximum rate temperature 510 °C and ends at a lower temperature than the others. The curve obtained for parsley root peels (Figure 9a, red curve) has a similar shape to that noted for the potato peel with a small wide exothermic peak at approximately 286 °C, followed by a much larger peak with the maximum thermal effect at 530 °C. Considering the fact that the ash composition of these two is similar, they could be used either as co-feed of a biomass energy generator or for biochar formation. The oats and willow have a slightly larger first signal, which occurs at 305 °C and 340 °C, respectively. This thermal effect is the smallest in the case of the field beans. In their case, the maximum of the thermal effect is observed at 605 and 600 °C. The maximum thermal effect during combustion of coal and field beans occurred at 595 °C. The weight loss curves measured during the co-firing experiments are shown in Figure 9b. The potato peels showed the smallest first loss step and fastest rate of combustion, which is seen in the steep slope of the second weight loss step. They also have the most ash, which is another reason to use parsley root peels as co-feed in order to reduce the amount of ash slag from such a power plant. Considering the fact that the willow exhibits a much different ash composition, with calcium carbonate being the main constituent rather than a potassium-containing compound, it could be added as co-feed to control the ash/slag composition for the application of such ash/slag in, e.g., cement production [48,49].

The evolution of water (*m*/*z* = 18) and CO_2_ (*m*/*z* = 44) during co-firing measurements was monitored. The curves are shown in Figure 9c,d, respectively. Although the first type of curve is very similar and exhibits the largest emissions of H_2_O in the temperature range between 300 °C and 350 °C, which is associated with the devolatilization of samples, the CO_2_ curves are interesting in that the amount of CO_2_ released during combustion (i.e., the area under the curve) is not the same for all samples. The much smaller emission of CO_2_ during the co-firing of oats and carbon than for all other samples may indicate incomplete combustion of the biomass. However, the samples were combusted in synthetic air, so the signals for CO and N_2_ overlap, and further studies with a different mixture, e.g., O_2_+Ar, would be needed to verify this hypothesis. Nevertheless, in the case of oats, such a result is a strong indication that they would be more suitable for soil remediation than for application in energy production.

## 4. Conclusions

This study delved into the idea of using biomass in one of two potential applications: energy generation or soil remediation. Although most studies have only dealt with one type of biomass, this paper encompassed six samples to allow for comparison between food production waste (potato peels and parsley root peels) and crops used for soil upgrading (which do not meet the standards of EU crops: oats, field beans) with the results obtained for basket willow, which is commonly used for energy generation. The common trait of these different feedstocks was that they are available on a large scale in a single-component stream, making implementation feasible. A novel type of pyrolytic reactor was used for charring of the biomass, which allowed for a complete transformation of biomass without combustion of char, despite the flow of ambient air through the reactor. An aluminum foil wrapper ensured that the devolatilization occurred under the atmosphere of the evolving gases; hence, the biochar obtained using the reactor was suitable for soil remediation. The composition of samples of the obtained biochar and ash was probed, and the results showed that there are differences in the composition of ash, which could allow for blending of the biomass to obtain ash/slag suitable for various applications. Experiments with the biomass used as a co-feed in combustion with coal were performed. The results showed substantial differences in onset and end temperatures, as well as CO_2_ evolution. The lowest temperatures were an indication of catalytic properties of ash present in the biomass samples and suggested application in energy generation, as in the case of potato peels and parsley root peels. In contrast, low CO_2_ levels in the combustion of the same weight (0.4 g) of the 1:1 biomass/carbon mixture showed that biochar production should be considered, such as in the case of oats. The composition of the ash indicated that the willow can be used for co-firing with all other types of studied biomass. 

## Figures and Tables

**Table 1:** Proximate analysis of biomass samples.

Biomass	Char + Ash (Pyrolytic Reactor)	Char + Ash (Muffle Furnace)	Ash
Basket willow	21.4	22.3	5.8
Parsley root peels	32.3	33.8	4.4
Oats	50.4	71.2	2.7
Potato peels	38.1	36.3	9.1
Field beans	35.4	40.1	3.6
Hay	33.7	33.4	7.6

## Data Availability

All data are available in the manuscript.

## Supplementary Materials

The following supporting information can be downloaded at: https://www.mdpi.com/article/10.3390/ma17010226/s1, Figure S1: STEM images of biochar derived from: (a) potato peels, (b) oats and (c) willow.
