# Supplementary figures and images for: Possible Options for Utilization of EU Biomass Waste: Pyrolysis Char, Calorific Value and Ash Content
Source: Materials (Basel). 2023 Dec 31;17(1):226. doi: 10.3390/ma17010226 (PMC10780033; doi:10.3390/ma17010226)

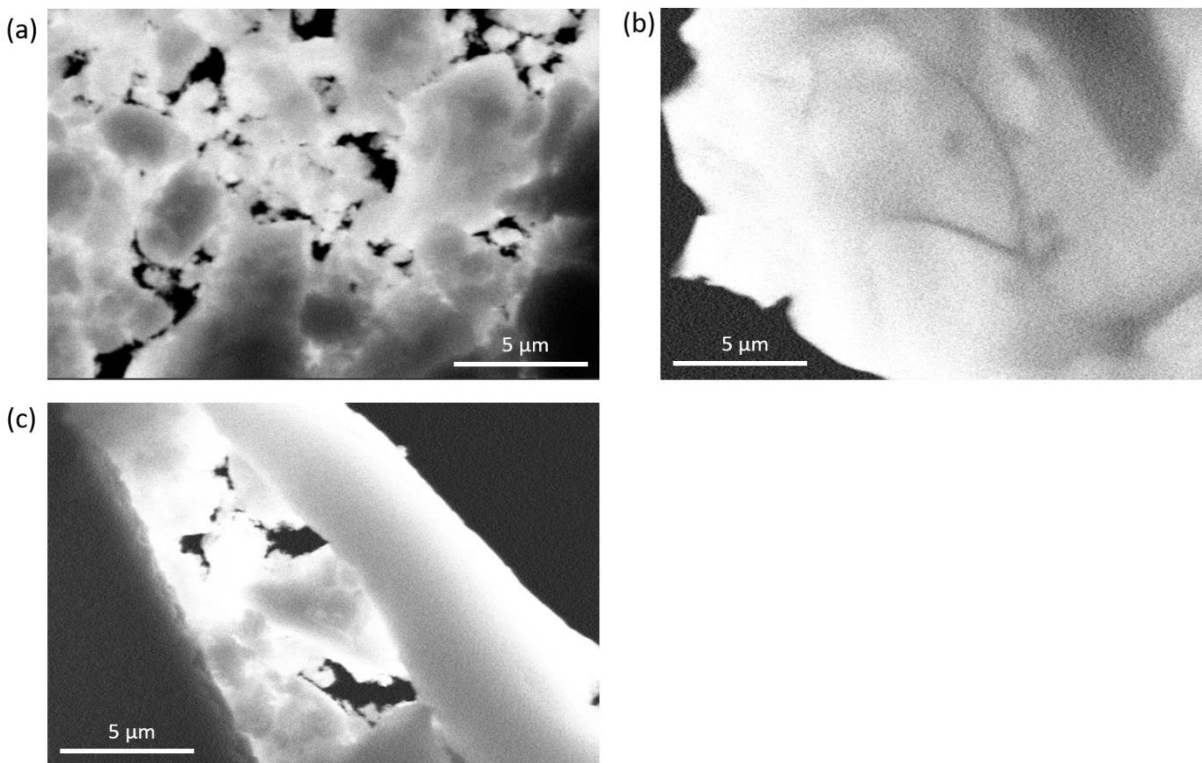

Figure S1: STEM images of biochar derived from: (a) potato peels, (b) oats and (c) willow.

Supplement: Supplementary file 1 [file materials-17-00226-s001.zip › materials-2746318-supplementary.pdf]
